# Endogenous GDF11 regulates odontogenic differentiation of dental pulp stem cells

**DOI:** 10.1111/jcmm.15754

**Published:** 2020-08-26

**Authors:** Xingying Qi, Qingyue Xiao, Rui Sheng, Shuang Jiang, Quan Yuan, Weiqing Liu

**Affiliations:** ^1^ State Key Laboratory of Oral Diseases National Clinical Research Center for Oral Disease West China Hospital of Stomatology Sichuan University Chengdu China; ^2^ Department of Oral Implantology West China Hospital of Stomatology Sichuan University Chengdu China

**Keywords:** GDF11, human dental pulp stem cells, odontogenic differentiation

## Abstract

Dental stem cell‐based tooth regeneration is the futuristic treatment for missing teeth. Growth differentiation factor 11 (GDF11), a novel member of the TGF‐beta superfamily, has been reported to play a critical role in regulating stem cell differentiation. However, the role of endogenous GDF11 during dental stem cell differentiation remains unknown. Here, we have shown that GDF11 was highly expressed in dental pulp tissues in both mouse and human. Knockdown of endogenous GDF11 in human dental pulp stem cells (hDPSCs) led to comparable proliferation and migration but attenuated odontogenic differentiation as evidenced by alkaline phosphatase and Alizarin Red S staining. In addition, transcriptional levels of odontogenic‐related genes were significantly down‐regulated according to real‐time polymerase chain reaction. Mechanistically, we performed RNA sequencing analysis and found that silencing of endogenous GDF11 compromised the process of ossification and osteoblast differentiation, especially down‐regulated transcription expression of Wnt pathway‐specific genes. Immunofluorescence staining also showed diminished β‐catenin expression and nuclei accumulation after knockdown of endogenous GDF11 in hDPSCs. In summary, our results suggested that endogenous GDF11 positively regulate odontogenic differentiation of hDPSCs through canonical Wnt/β‐catenin signalling pathway.

## 1 INTRODUCTION

Tooth defect and tooth loss are growing in prevalence worldwide, driven by increasing caries and periodontal diseases. Current clinical dentistry is predominantly focused on restorative approaches using inorganic materials, while tissue regeneration is an attractive alternative since inorganic materials alone fail with time and cannot completely restore the physiological function of tooth.^1^ Several physiological processes are being investigated to explore novel methods of stem cell‐based tooth regeneration.^2^, ^3^, ^4^ Upon extensive tooth injury and pulp exposure, quiescent dental pulp stem cells (DPSCs) inhabiting around pulp blood vessels would be activated and migrate towards the trauma site and refresh odontoblasts to form reparative dentine.^5^, ^6^ Among multiple growth factors mediating this process, bone morphogenetic protein (BMP)/transforming growth factor‐β (TGF‐β) superfamily is being increasingly recognized as a group of potent stimulators for DPSCs recruitment, proliferation and odontogenic differentiation.^3^, ^7^, ^8^, ^9^

Growth differentiation factor 11 (GDF11), also known as bone morphogenetic protein 11 (BMP11), is a novel member of BMP/ TGF‐β superfamily originally identified from rat incisor dental pulp RNA.^10^, ^11^ Whole‐mount RNA in situ hybridization analysis showed that *Gdf11* was highly expressed in mesodermal tissues of early mouse embryos. The specific role of GDF11 in bone development has been demonstrated in knockout mice that display skeletal patterning problems related to anterior‐posterior positioning.^12^, ^13^ Our previous work showed that injection of recombinant GDF11 resulted in bone loss, as well as inhibition of bone regeneration in both young and aged mice, suggesting the negative role of exogenous GDF11 in osteogenesis during postnatal bone remodelling.^14^

As mineralized tissue, tooth and bone share many similarities in cell maturation and mineralization.^15^, ^16^ During tooth development, *Gdf11* expression was first detected in preodontoblasts at the late cap stage and rose gradually till terminally differentiated odontoblasts on first day after birth,^10^ indicating an possible role of GDF11 in tooth development. As to mature tooth, gene delivery of exogenous GDF11 could induce DPSCs differentiation and reparative dentine formation.^17^, ^18^, ^19^ What has not been fully elucidated is the effects and underlying mechanisms of endogenous GDF11 in dental stem cell differentiation. As a result, we sought to investigate whether and how endogenous GDF11 could play a regulatory role in odontogenetic differentiation.

## 2 MATERIALS AND METHODS

### 2.1 Tissue collection

Healthy human third molars undergoing tooth extraction were obtained according to the informed protocol approved by the Committee on Human Research of the West China Hospital of Stomatology of Sichuan University (Approval No. WCHSIRB‐D‐2017‐201). Mouse mandibles were harvested from 8‐week‐old male C57BL/6 mice. For frozen sections, human molars and mouse mandibles were fixed in 4% paraformaldehyde and decalcified in 10% EDTA at 4°C.

### 2.2 Isolation and culture of primary cell

Third molars were obtained from healthy patient under 25 years old at West China Hospital of Stomatology of Sichuan University, with informed consent. After washing twice in PBS, dental pulp tissues were retrieved in sterile conditions, cut into as small fragments as possible and digested in 0.5% type I collagenase for 30 minutes at 37°C. Then, tissue fragments were terminated digestion in fresh α‐minimal essential medium (α‐MEM) containing 10% heat‐activated foetal bovine serum (FBS, all from Gibco). Tissues and cells were precipitated by centrifuging at 230 *g* for 5 minutes, then resuspended with α‐MEM growth medium and transferred to dishes. Human dental pulp stem cells (hDPSCs) were maintained in α‐MEM containing 10% foetal bovine serum (FBS) and 1% penicillin/streptomycin (PS) at 37°C in moist atmosphere with 5% CO_2_ for use. For odontogenic induction, hDPSCs were cultured in odontogenic media (OM) containing 100 µmol/L ascorbic acid, 2 mmol/L β‐glycerophosphate, and 10 nmol/L dexamethasone (all from Sigma).

### 2.3 Endogenous GDF11 silencing by siRNA

Human GDF11 and control small interfering RNAs (siRNAs) were purchased from Santa Cruz. After incubating with Lipofectamine RNA iMAX (Life technologies)‐siRNA duplex for 35 minutes at room temperature, control siRNA or GDF11 siRNA was transfected into hDPSCs.

### 2.4 Quantitative real‐time polymerase chain reaction

Total RNA was isolated from hDPSCs using Trizol (Invitrogen). The acquired messenger RNA (mRNA) underwent reverse transcribed to synthesize complementary DNA (cDNA) by TaqMan Reverse Transcription Reagents (Takara). The sequences were amplified and measured by quantitative real‐time polymerase chain reaction (RT‐PCR) using SYBR Green PCR system according to manufacturer instructions. All samples were normalized to* GAPDH*. The following primer sets were used: *GDF11*, 5′‐CAAGTCGCAGATCTTGAGCA‐3′ (sense) and 5′‐CACTTGCTTGAAGTCGATGC‐3′ (antisense); *RUNX2*, 5′‐TGGTTACTGTCATGGCGGGTA‐3′ (sense) and 5′‐TCTCAGATCGTTGAACCTTGCTA‐3′ (antisense); *DSPP*, 5′‐ATATTGAGGGCTGGAATGGGGA‐3′ (sense) and 5′‐TTTGTGGCTCCAGCATTGTCA ‐3′ (antisense); *OCN*, 5′‐CACTCCTCGCCCTATTGGC‐3′ (sense) and 5′‐CCCTCCTGCTTGGACACAAAG‐3′ (antisense); *COL1a1*, 5′‐GTGCGATGACGTGATCTGTGA‐3′ (sense) and 5′‐CGGTGGTTTCTTGGTCGGT‐3′ (antisense); *AXIN2*, 5′‐ACCCTGGGCCACTTTAAAG‐3′ (sense) and 5′‐CCTTCATACATCGGGAGCAC‐3′ (antisense); *TCF1*, 5′‐ACCAAGAATCCACCACAGGAG‐3′ (sense) and 5′‐CGCAGGGCTAGTAAGCAGTT‐3′ (antisense); *TCF4*, 5′‐CCTGGCACCGTAGGACAAAT‐3′ (sense) and 5′‐GGAACCTGGACATGGAAGCA‐3′ (antisense); *LEF1*, 5′‐CTGCATCAGGTACAGGTCCA‐3′ (sense) and 5′‐AGAGGGGTTGGCAGTGATTG‐3′ (antisense); *DKK2*, 5′‐ACCATGACTTGGGATGGCAG‐3′ (sense) and 5′‐CCCTGATGGAGCACTGGTTT‐3′ (antisense); *SOST*, 5′‐AACAAGACCATGAACCGGG‐3′ (sense) and 5′‐GCAGCTGTACTCGGACAC‐3′ (antisense); *GAPDH*, 5′‐ATCACTGCCACCCAGAAGAC‐3′ (sense) and 5′‐ATGAGGTCCACCACCCTGTT‐3′ (antisense).

### 2.5 Alkaline phosphatase (ALP) activity and alizarin red s (ARS) staining

For ALP staining, hDPSCs were induced in odontogenic media (OM) for 14 days. Then, cells were fixed with 4% paraformaldehyde and incubated with a solution of 0.25% naphthol AS‐BI phosphate and 0.75% fast blue dissolved in 0.1 mol/L Tris buffer (pH 9.3). ALP activity was quantified using a commercial kit according to the manufacturer’s protocol (Cell Biolab).

To detect mineralized nodules, cells were induced for 21 days, fixed with 4% paraformaldehyde, and stained with 2% Alizarin Red S according to manufacturer instructions (pH 4.2, Sigma‐Aldrich). Mineralized nodules stained with alizarin red were eluted with 10% cetylpyridinium chloride in 10 mmol/L sodium phosphate (pH 7.0) and determined by absorbance measurements at 562 nm using a standard calcium curve in the same solution.

### 2.6 Cell proliferation and migration analysis

The impact of endogenous GDF11 knockdown on the proliferation of hDPSCs was examined using the Cell Counting Kit‐8 (CCK‐8; MCE). Briefly, hDPSCs transfected with either the siGDF11 or siCTRL liposome were seeded into three 96‐well plates at a density of 3 × 10^3^ cells/well and then cultured overnight. Cell proliferation was then examined on days 3, 5 and 7. The CCK‐8 reagent (10 mL) was added into each well, and the cells were incubated for 2 hours at 37°C in the dark. Absorbance at 450 nm was measured on a microplate reader (PerkinElmer) to determine the number of live cells in each well.

For wound healing evaluation, hDPSCs were seeded in 6‐well plates and grown at 37°C until 100% confluence. The cell monolayers were scratched with a 200 µL sterile pipette tip to create a wound. Then, the cell monolayers were washed three times with PBS and were cultured for additional 24 hours in 1.5 mL of the serum‐free growth medium per well. The migration of the cells across the wound line was assessed using brightfield microscopy.

### 2.7 RNA sequencing

Expression levels of mRNA in hDPSCs pre‐treated with siGDF11 or siCTRL were measured by RNA Sequencing after 7 days of odontogenic induction. Total RNA was isolated using a RNeasy kit (Qiagen) according to the manufacturer's protocol. Transcriptome profiling was performed in triplicate using human gene 1.0 ST Array. Expression data were analysed using Transcriptome Analysis Console 3.0 software. Heatmap for the Wnt pathway‐related specific genes were drawn with Morpheus. The threshold for differentially regulated transcripts was set as the fold change of 1.5 with a *P* value of <.05.

### 2.8 Immunofluorescence

For frozen sections, decalcified tooth and mandibles were sectioned at 8 µm thickness. For cellular coverslips, hDPSCs were grown to 50% confluence, then transfected with siRNA for 48 hours. Glass coverslips with cells were washed three times with PBS and fixed with acetone for 20 minutes at room temperature. The cells were permeabilized with 0.5% Triton‐X for 10 minutes and blocked in blocking buffer (1%BSA/ 5% normal goat serum) for 30 minutes at room temperature. The frozen sections and the cellular coverslips were incubated overnight with appropriate primary antibodies anti‐GDF11 (1:500, #MAB 19581, R&D System) and anti‐β‐catenin (1:500, #51067‐2‐AP, Proteintech), respectively, at 4°C. This procedure was followed by secondary antibody fluorescent‐labelling with Alexa Fluor 550 for 1 hour at room temperature. The cell nuclei were also labelled with diamidino‐phenyl‐indole DAPI. Images were obtained using fluorescence confocal microscope.

## 3 RESULTS

### 3.1 Expression of GDF11 in human and mouse dental pulp tissue

**FIGURE 1:**
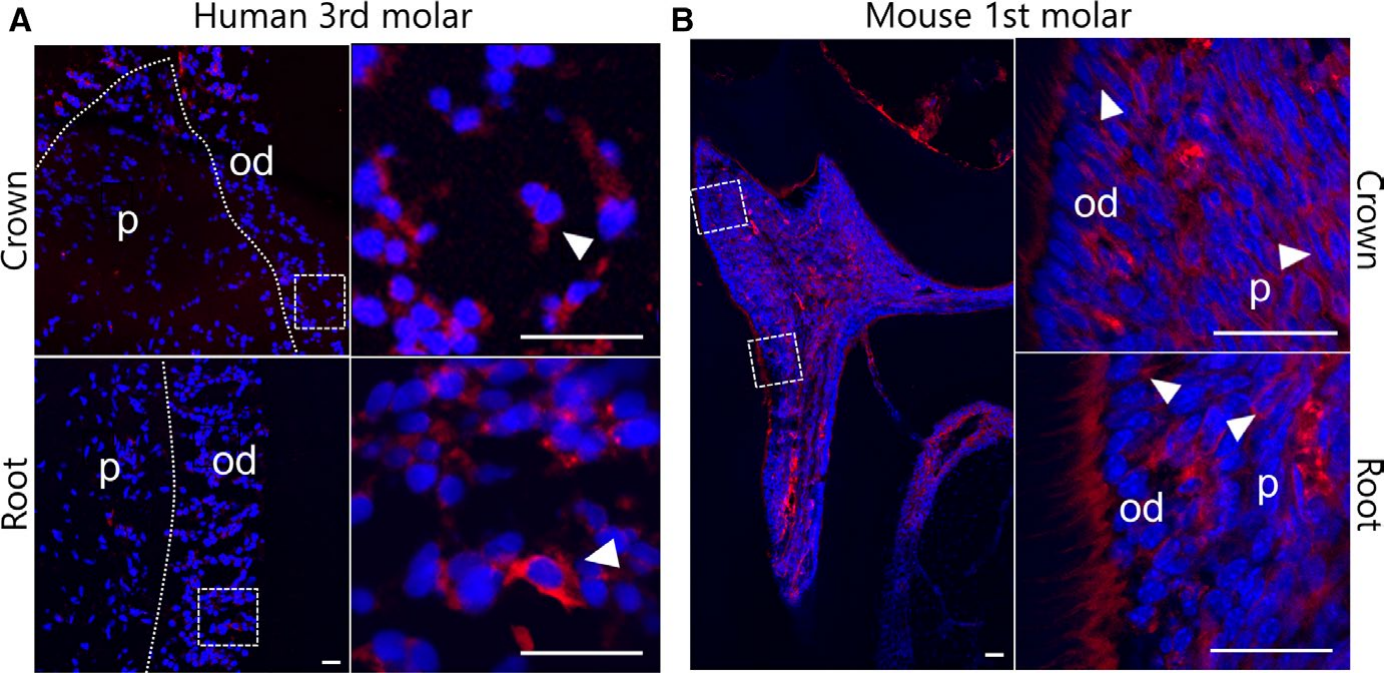
Expression of GDF11 in human and mouse dental pulp tissue. A, Expression of GDF11 in mature human third molar. By immunofluorescence staining, GDF11 expression could be detected in the cytoplasm of both crown and root pulp cells, with especially high level in the outermost odontoblast layer (white frame). The white dotted lines represent the boundary between the odontoblast layer and the inner pulp. B, Expression of GDF11 in mouse first molar. GDF11 is detected in the cytoplasm of all dental cells in mouse. Higher magnification view of the white frame is shown on the right. Arrowheads indicate representative GDF11 expression (red) in the cytoplasm. Nuclei are counterstained with DAPI (blue). Scale bars: 50 µm (left panels), 20 µm (right panels). p, pulp; od, odontoblast

To explore the expression of endogenous GDF11 in mature tooth, immunofluorescence staining of GDF11 was performed in both healthy human third molar and mouse first molar. GDF11‐positive staining was detected in part of human pulp tissue and notably obvious in the odontoblast layer outmost of pulp (Figure 1A). While in mouse pulp, GDF11 was highly expressed in the whole pulp tissue including odontoblast layer (Figure 1B).

### 3.2 Depletion of endogenous GDF11 impairs the differentiation of hDPSCs

**FIGURE 2:**
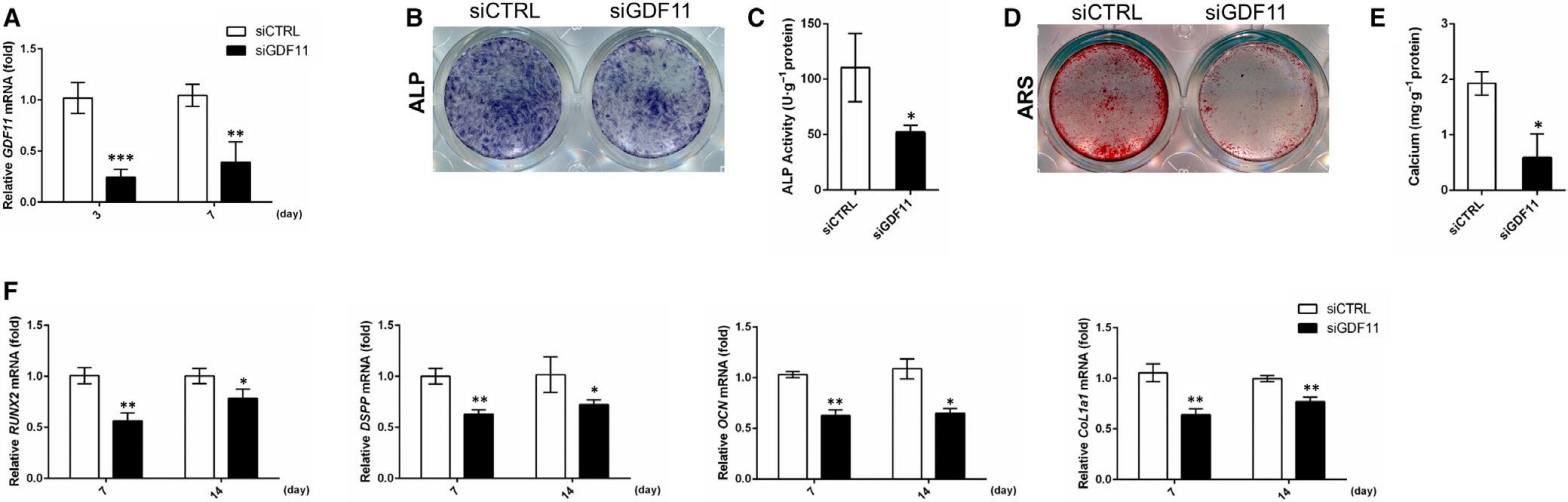
Depletion of endogenous GDF11 impairs odontogenic differentiation of hDPSCs. A, Quantitative real‐time polymerase chain reaction (RT‐PCR) analysis of knockdown efficiency of GDF11 siRNA. Endogenous GDF11 is successfully knockdown by over 70%. B‐E, ALP activity assay and ARS staining of hDPSCs. The hDPSCs transfected with GDF11 siRNA show a significant decrease in ALPase activity after osteogenic induction for 14 d (B, C) as well as mineralized deposits after osteogenic induction for 21 d (D, E). F, Quantitative RT‐PCR analysis of odontogenic markers expression after 7 and 14 d of odontogenic differentiation induction. Knockdown of endogenous GDF11 significantly decreases odontogenic markers (*RUNX2, DSPP, OCN and COL1a1*) expression. Data are presented as mean ± SD of 3 independent experiments. *P < .05, **P < .01, ***P < .001 by *t* test

siRNA was transfected into hDPSCs and able to knock down endogenous GDF11 by over 70% (Figure 2A). When cultured in odontogenic medium, hDPSCs transfected with siCTRL underwent odontogenic differentiation and mineralization as demonstrated by distinct ALP staining and ARS staining (Figure 2B, D). While knockdown of endogenous GDF11 significantly attenuated ALP activity and mineralization of hDPSCs after odontogenic differentiation up to 14 and 21 days, respectively (Figure 2B‐E). In addition, quantitative RT‐PCR was performed to examine the mRNA expression of dentin‐related markers, runt‐related transcription factor 2 (*RUNX2*), dentin sialophosphoprotein (*DSPP*), osteocalcin (*OCN*) and collagen type I alpha 1 chain (*COL1a1*). The transcription levels of these genes in hDPSC transfected with siGDF11 were down‐regulated when compared with siCTRL cells at each time point (Figure 2F), which was consistent with attenuated ALP activity and mineralized deposits.

### 3.3 GDF11 knockdown does not affect proliferation or migration of hDPSCs

**FIGURE 3:**
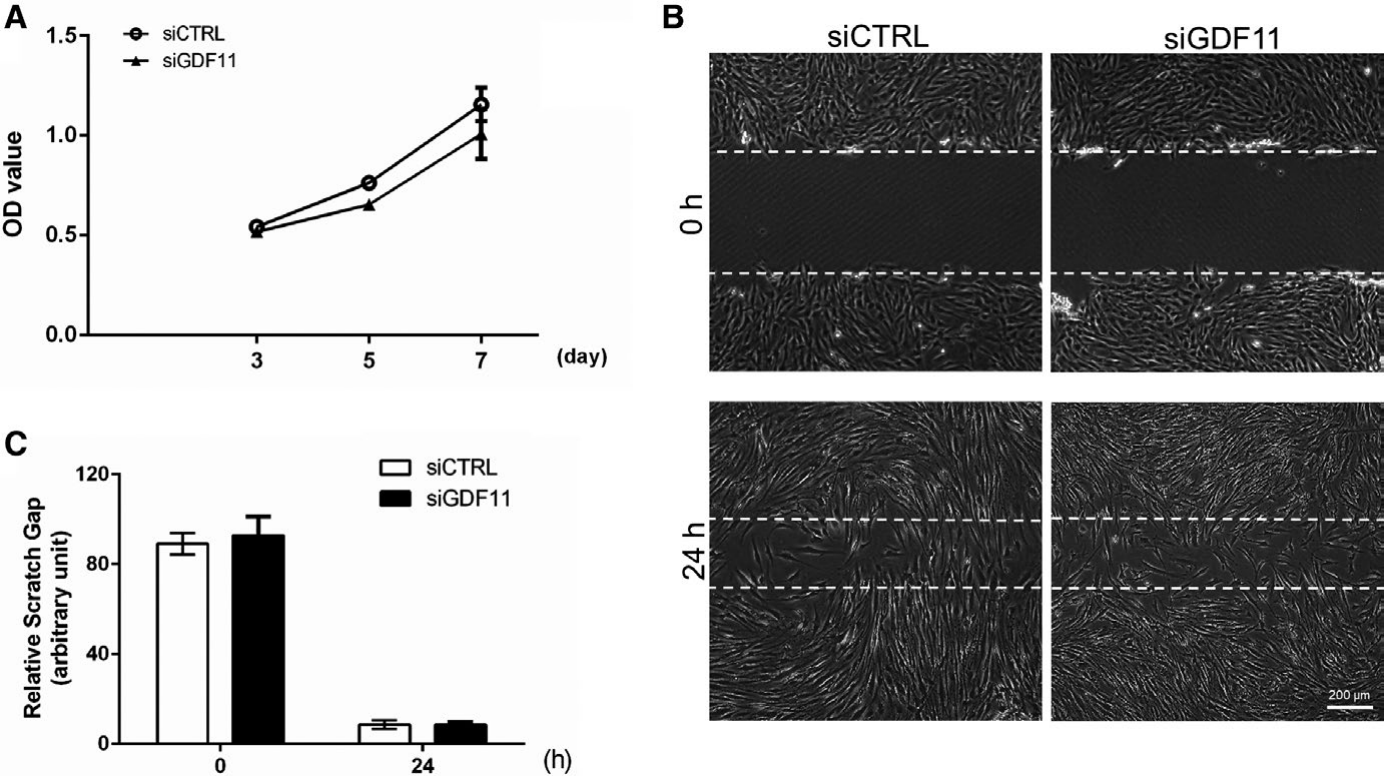
Knockdown of GDF11 does not affect proliferation and migration of hDPSCs. A, Quantitative analysis of cell proliferation in 3, 5 and 7 d after endogenous GDF11 knockdown. There is no significant difference between siGDF11 group and control group as shown by Cell Counting Kit‐8 (CCK8) assay. B and C, Quantitative analysis of scratch wound healing in hDPSCs. No significant difference is observed between hDPSCs transfected with siGDF11 and siCTRL. Data are presented as mean ± SD of 3 independent experiments. **P < .01 and ***P < .001 by *t* test

The cell proliferation assay was performed to assess the effects of endogenous GDF11 on cell viability of hDPSCs. After transfected with siGDF11, hDPSCs showed comparable proliferation rate to that of control group (Figure 3A). In parallel, the scratch wound healing assay demonstrated no significant difference between siGDF11‐ and siCTRL‐transfected hDPSCs (Figure 3B, C). Taken together, knockdown of endogenous GDF11 had little effect on cell proliferation and migration, which exclude the possible effects of proliferation and migration on the promotion of hDPSCs differentiation and mineralization caused by endogenous GDF11.

### 3.4 Endogenous GDF11 knockdown inhibited the Wnt/β‐catenin signalling pathway

**FIGURE 4:**
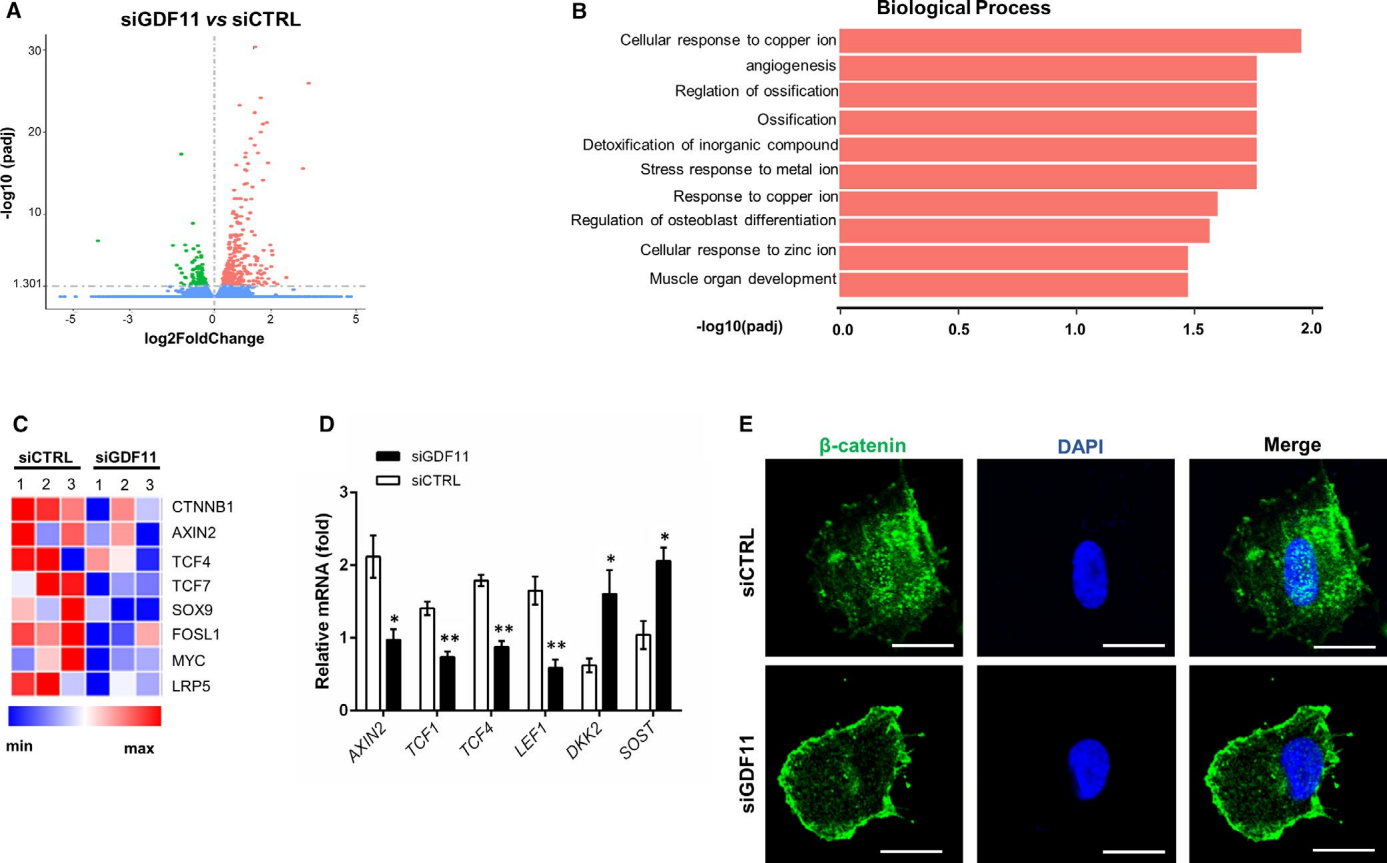
Knockdown of GDF11 inhibits Wnt/β‐catenin signalling pathway. A, Volcano plot of RNA‐seq data from hDPSCs with 7 d of odontogenic differentiation. Each point represents an individual gene and all genes differentially expressed in siGDF11 samples with an FDR of 0.05 are highlighted in red (up‐regulated) or green (down‐regulated). B, The ten most significant Gene Ontology terms in the Biological Processes category are shown for down‐regulated differentially expressed genes. C, Heatmap of representative genes involved in Wnt pathway. D, Quantitative RT‐PCR analyses of Wnt‐related genes in hDPSCs. Knockdown of endogenous GDF11 significantly down‐regulates the Wnt pathway reporter (*AXIN2*) and downstream transcriptional factors (*TCF1*, *TCF4* and *LEF1*), and up‐regulates the Wnt pathway inhibitors (*DKK2* and *SOST*). Results are expressed as relative expression to *GAPDH*. E, Immunofluorescence staining of β‐catenin in hDPSCs. hDPSCs transfected with siGDF11 exhibits decreased β‐catenin expression and attenuated translocation into the nucleus. Scale bars = 20 µm. Data are presented as mean ± SD of 3 independent experiments. *P < .05, **P < .01, ***P < .001 by *t* test

To gain insights into the transcriptional profiles that underlie the down‐regulation of odontogenic differentiation observed in siGDF11‐transfected hDPSCs, we performed RNA‐seq analysis on hDPSCs transfected with siGDF11 or siCTRL after 7 days of odontogenic induction. 445 differentially expressed genes (FDR < 0.05) were identified in siGDF11‐transfected hDPSCs, with about one third of these genes down‐regulated (Figure 4A). Among the down‐regulated gene set, the most significant biological processes were related to cellular response to ions, angiogenesis and especially ossification as well as osteoblast differentiation (Figure 4B). Notably, specific genes of canonical Wnt/β‐catenin signalling pathway were suppressed in siGDF11‐transfected hDPSCs, including transduction gene like catenin beta 1 (*CTNNB1*), reporter gene axis inhibition protein 2 (*AXIN2*), transcription factors like T‐cell factor (*TCF*)‐4, 7, low‐density lipoprotein receptor‐related protein 5 (*LRP5*) receptors and so on (Figure 4C).

To further confirm these findings, we performed quantitative RT‐PCR analysis and found that hDPSCs transfected with siGDF11 showed significant decreased transcription of *AXIN2* and several downstream targets, such as *TCF1*, *TCF4* and lymphoid enhancer‐binding factor 1 (*LEF1*) after 14 days of odontogenic induction. (Figure 4D). While the expression of inhibitors in Wnt pathway, dickkopf‐related protein 2 (*DKK2*) and sclerostin (*SOST*), was inversely up‐regulated after knockdown of endogenous GDF11 in hDPSCs (Figure 4D). Immunocytochemical staining of β‐catenin in hDPSCs transfected with control siRNA showed high β‐catenin expression and its accumulation in the nuclei during odontogenic differentiation, whereas siGDF11 transfection significantly diminished both β‐catenin expression in cytoplasm and its translocation into the nuclei (Figure 4E).

## 4 DISCUSSION

The reparative dentine is formed after severe tooth lesions and serves as the barrier to protect exposed pulp against external damages.^5^ But naturally formed thin bridge of reparative dentine is insufficient to restore the lesion especially after caries removal. Stem cell‐based therapies are called for to regenerate the lost dentine and/or dental pulp tissues, replacing or assisting traditional filling methods.^4^

BMP/TGF‐β superfamily is widely expressed in multiple tissues and organs such as tooth and bone, and plays a pivotal role in tissue regeneration and homeostasis.^20^ Since GDF11, an emerging member of BMP/ TGF‐β superfamily, was first found in 1999,^10^ a number of researches have focused on its role in bone development and remodelling,^14^, ^21^ but few studies investigated its effects on tooth. Nakashima and his colleagues have demonstrated that gene delivery of exogenous GDF11 by electroporation or ultrasound could positively regulate DPSCs differentiation.^17^, ^18^, ^19^ There is still a lack of research on endogenous GDF11 during DPSCs differentiation as the early lethality of *Gdf11* loss‐of‐function mutations.^12^, ^22^

In this study, we have unveiled the positive role of endogenous GDF11 in hDPSCs differentiation. Initially, our ex vivo data revealed that GDF11 was widely expressed in dental pulp tissues both in mature human and mouse molars. Of note, the expression of GDF11 was significantly higher in the odontoblastic layer than in the central part of human dental pulp. Accompanied with the fact that robust expression in preodontoblasts at cap and bell stage of tooth germ,^10^ it is hypothesed that endogenous GDF11 could play a part in the differentiation of DPSCs into odontoblasts. To this end, quantitative RT‐PCR was utilized to examine responses of differentiation‐related genes to suppressing endogenous GDF11. The expression of *OCN* and *DSPP*, specific markers of dentinogenesis, was significantly decreased in hDPSCs transfected with siGDF11. The general markers in the early and late stage of osteogenesis, such as *RUNX2* and *COL1a1*, were also down‐regulated after knockdown of endogenous GDF11. ALPase activity and ARS were further performed to evaluate the ability of early osteogenesis and late mineralization, which were attenuated in line with the results of quantitative RT‐PCR after endogenous GDF11 knockdown. These findings suggested that endogenous GDF11 could promote odontogenic differentiation of hDPSCs.

In addition to differentiating into odontoblasts, proliferation and migration of the resident DPSCs are needed to effectively form new dentin matrix. GDF11 has been reported to regulate proliferation and migration of endothelial cells in various tissues and progenitor cells during early axial development.^23^, ^24^ In the present study, we knock down the expression of endogenous GDF11 to investigate its effect on proliferation and migration of hDPSCs. Compared with control group, suppressed expression of endogenous GDF11 had no significant effect on hDPSCs proliferation and migration, suggesting the specific role of endogenous GDF11 in regulating hDPSCs differentiation and mineralization without necessarily affecting cell motility or growth.

The down‐regulation of odontogenic differentiation by siGDF11 was further confirmed by RNA sequencing analysis, with significantly decreased processes of ossification and osteoblast differentiation. Accumulating evidence has supported the association between dentine mineralization and canonical Wnt/β‐catenin signalling pathway.^25^, ^26^, ^27^, ^28^, ^29^, ^30^, ^31^ β‐catenin has been demonstrated to be expressed in odontoblastic layer adjacent to dentine, its nuclei translocation could notably promote the proliferation and differentiation of DPSCs,^28^, ^32^, ^33^, ^34^ indicating that Wnt/β‐catenin signalling pathway might be involved in the effect of endogenous GDF11 on hDPSCs. Consistently, knockdown of endogenous GDF11 did result in the down‐regulated transcription expression of Wnt pathway‐specific genes as shown in RNA‐seq data. The involvement of Wnt/β‐catenin pathway was further validated in the late stage of odontogenic differentiation (14 days), with suppressed transcription of several representative Wnt‐related genes, such as *AXIN2*, *TCF1*, *TCF4* and *LEF1*, and elevated expression of negative regulators like *DKK2* and *SOST* after knockdown of endogenous GDF11. In addition, the immunocytochemical staining of β‐catenin showed that silencing of endogenous GDF11 resulted in a decrease in cytoplasmic β‐catenin expression and diminished nuclei translocation, suggesting that endogenous GDF11 induce odontogenic differentiation of hDPSCs partly via canonical Wnt/β‐catenin signalling pathway. Studies have shown that the differentiation of dental pulp stem cells can be regulated by multiple pathways, such as TGF‐β and p38 MAPK signalling pathway.^2^, ^3^, ^5^, ^7^ Therefore, we could not rule out the possible role of endogenous GDF11 in the regulation of other Wnt‐related genes or other signalling pathways during odontogenic differentiation of hDPSCs.

As many other members of BMP/ TGF‐β superfamily, GDF11 is initially synthesized in a pattern of precursors with prodomain sequences, followed by a proteolytic cleavage to generate the functional C‐terminal peptide or mature GDF11 protein, and the N‐terminal peptide called GDF11 propeptide.^11^ It has been documented that the cleaved propeptide was capable of forming a latent complex with GDF11 and act as a negative feedback regulator for GDF11 function in vitro and ex vivo.^11^, ^35^, ^36^, ^37^ Given the ambiguity of self‐regulated effects in GDF11, overexpression of GDF11 was not performed to assess whether this could rescue the attenuated odontogenic differentiation of hDPSCs.

In conclusion, our findings illustrated the positive role of endogenous GDF11 on the odontogenetic differentiation of hDPSCs, which was mediated by the activation of canonical Wnt/β‐catenin signalling pathway. It is noteworthy that our study provided a novel insight into the biological function of endogenous GDF11 during dentinogenesis. Further investigations and attempts are warranted to interpret the comprehensive mechanisms of endogenous GDF11 in tooth regeneration.

## CONFLICT OF INTEREST

We declare that we have no financial and personal conflict of interests.

## AUTHOR CONTRIBUTIONS

**Xingying Qi:** Conceptualization (lead); Data curation (lead); Formal analysis (lead); Investigation (lead); Methodology (lead); Software (lead); Validation (lead); Visualization (lead); Writing‐original draft (lead); Writing‐review & editing (lead). **Qingyue Xiao:** Methodology (equal); Validation (equal). **Rui Sheng:** Methodology (equal); Validation (equal). **Shuang Jiang:** Methodology (equal); Validation (equal). **Quan Yuan:** Funding acquisition (supporting); Supervision (supporting). **Weiqing Liu:** Conceptualization (supporting); Funding acquisition (lead); Resources (lead); Supervision (lead).

## Data Availability

RNA‐seq that support the findings of this study have been deposited in NCBI GEO with the identifier GSE151435. The data that support the findings of this study are available from the corresponding author upon reasonable request.
